# Quantification of Polychlorinated Biphenyls and Polybrominated Diphenyl Ethers in Commercial Cows’ Milk from California by Gas Chromatography–Triple Quadruple Mass Spectrometry

**DOI:** 10.1371/journal.pone.0170129

**Published:** 2017-01-13

**Authors:** Xiaopeng Chen, Yanping Lin, Katherine Dang, Birgit Puschner

**Affiliations:** Department of Molecular Biosciences, School of Veterinary Medicine, University of California, Davis, California, United States of America; University of Iowa, UNITED STATES

## Abstract

We determined 12 polybrominated diphenyl ethers (PBDEs) and 19 polychlorinated biphenyls (PCBs) congeners in eight different brands of commercial whole milk (WM) and fat free milk (FFM) produced and distributed in California. Congeners were extracted using a modified quick, easy, cheap, effective, rugged and safe (QuEChERS) method, purified by gel permeation chromatography, and quantified using gas chromatography-triple quadruple mass spectrometry. PBDEs and PCBs were detected in all FFM and WM samples. The most prevalent PBDE congeners in WM were BDE-47 (geometric mean: 18.0 pg/mL, 0.51 ng/g lipid), BDE-99 (geometric mean: 9.9 pg/mL, 0.28 ng/g lipid), and BDE-49 (geometric mean: 6.0 pg/mL, 0.17 ng/g lipid). The dominant PCB congeners in WM were PCB-101(geometric mean: 23.6 pg/mL, 0.67 ng/g lipid), PCB-118 (geometric mean: 25.2 pg/mL, 0.72 ng/g lipid), and PCB-138 (geometric mean: 25.3 pg/mL, 0.72 ng/g lipid). The sum of all 19 PCB congeners in FFM and WM were several orders of magnitude below the U.S. FDA tolerance. The sum of PBDEs in milk samples suggest close proximity to industrial emissions, and confirm previous findings of elevated PBDE levels in California compared to other regions in the United States.

## Introduction

Dairy foods provide a variety of essential minerals, vitamins, and proteins known to be beneficial for health. However, exposure to potentially deleterious compounds through milk and dairy products must be considered as an important public health issue. Commercial cow milk is a source for exposure to polychlorinated biphenyls (PCBs) and polybrominated diphenyl ethers (PBDEs). PCBs and PBDEs are two groups of contaminants of particular concern because both are endocrine disruptors and neurotoxicants that persist and bioaccumulate due to their inherent high lipophilicity (log K_ow_ values range from 3 to 9). The chemical families of PBDEs and PCBs each consist of 209 congeners, many of which have been detected in human samples [1], raising concerns about their impacts on human health. Beginning in the 1920s PCBs were widely used as electrical insulators in transformers, capacitors, and heat exchangers, and as stabilizers in paints, plastics, and rubber products [2, 3]. PCBs were manufactured as mixtures of various congeners through progressive chlorination until a certain target percentage of chlorine by weight was achieved. In the U.S., these mixtures were referred to as Aroclors. The U.S. banned commercial PCB production in 1979. Major production of PBDEs began in the early 1970s for use as flame retardants in electronics, home furnishings, and foam products, including pet toys and bedding [4]. PBDE mixtures were produced commercially at three different levels of bromination, leading to the terms penta-, octa- and deca-BDEs. BDE-99, BDE-47, BDE-100, BDE-153 and BDE-154 are most commonly added to polyurethane foam used for furniture cushions as commercial penta-BDEs mixtures. Octa-BDEs are used in the plastic housing for televisions, computers and other electronics, and are composed of 70–80% of hepta- and octa-BDEs. Deca-BDEs, mainly containing BDE-209, are also added to high-impact plastic housing for electronic equipment, plastic furniture and plastic toys.[5]. In 2004, two commercial formulations, penta-BDE and octa-BDE, were banned or phased out of production in some U.S. states after a voluntary agreement between the U.S. EPA and the sole manufacturer of these products [6]. California, who was the first state to phase out penta- and octa-BDEs, banned production, processing and distribution of products containing more than 10 percent penta- or octa-BDEs through California Assembly Bill 302, which became effective January 2008. The bill exempts deca-BDEs and does not require labeling of PBDE-containing products [7].

PBDEs and PCBs are known to have adverse effects on the nervous and endocrine systems [8]. Attention has been directed toward ortho-poor PCB congeners (i.e., tetra- through octachlorobiphenyls with single or no ortho-chlorine atoms) because they can assume a coplanar configuration and interact with the aryl hydrocarbon receptor (AhR), mimicking the action of 2,3,7,8-tetrachlorodibenzo-*p*-dioxin (TCDD), despite generally low environmental occurrences [9–11]. Endocrine effects of the ortho-rich PCBs (congeners with 2–4 ortho-chlorine atoms) include weak estrogenicity, disruption of the hypothalamo-pituitary-thyroid axis, enhanced insulin secretion, and increased release of arachidonic acid [12–15]. Mechanistic studies of ortho-substituted PCB congeners have implicated these chemicals in neurotoxic responses [16–18]. Animal studies have demonstrated developmental neurotoxicity and alteration of thyroid hormone homeostasis after exposure to penta-BDEs, which consist of tetra- to hexa-congeners [19, 20]. In particular, rats and rabbits had decreased body weight, reduced ossification of bones, and morphological changes in liver and kidneys [20]. Deca-BDE mixtures (octa-to deca-BDEs) have low acute toxicity and are not known to be mutagenic or carcinogenic. The different toxicities of penta-, octa- and deca-BDEs can be, in part, attributed to differences in toxicokinetics, because higher brominated BDEs like deca-BDEs partition to a larger degree into adipose tissue and have lower rates of metabolism and elimination compared to penta- or octa-BDEs [19]. *In vitro* studies have shown differences in biotransformation between BDE-47 and BDE-99 using human liver microsomes [21, 22]. Animal studies have shown that highly brominated PBDE congeners, such as deca-BDEs, can be de-brominated and metabolized to lower brominated congeners [23–25] leading to increased levels of lower brominated BDE congeners *in vivo*. This is also illustrated by the observation that higher brominated PBDEs have shorter biological half-lives [26]. While there are no data on metabolism of PBDEs in bovine, BDE-99 has been shown to be de-brominated to BDE-49 in Chinook Salmon[27]. BDE-99 was also shown to be more extensively metabolized than BDE-47 in rats[28]. It is well established that biotransformation pathways are influenced by many biological factors, including species, age, hormone status, pregnancy, preexisting health conditions, and genetic polymorphisms[29].

California produces about 20.9% of the total milk in the United States, designating it the top dairy state in the U.S. While studies have been conducted in various countries in recent years to evaluate the concentrations of PBDEs and PCBs in milk and dairy products [30–33], very few such data exist for the U.S. [34], and no U.S. state-specific data have been established. Assessing the concentrations of PBDEs and PCBs in milk and dairy products from California is a much needed study. Soil, silage, hay and pasture can become polluted by PCB and PBDE contaminated sewage sludge run off from waste water treatment plants, older coatings of silos and air-soil deposition from industrial emissions [35–37]. Fugacity models depicting vegetation-to-milk, air-to-grass, and grass silage-to-lactating dairy cow transfer rates have shown that contaminated feed represents the greatest source for PCBs and PBDEs in lactating cows, while transfer via air and water is almost negligible [38]. Once absorbed, PCBs and PBDEs are rapidly distributed to tissues and milk. In fact, secretion of lipophilic compounds into milk is a major pathway for elimination and is influenced by the animal’s sex, age and physiological state of production [39, 40].

We determined PCB and PBDE concentrations in store-bought bovine milk distributed by eight commercial producers in the state of California. Based on the proportions in commercial mixtures and toxicity data, we selected and quantified 19 PCB congeners including indicator PCBs 101, 138, 153 and 180 [41] and 12 PBDE congeners, mainly penta- and octa- BDEs. The region-specific evaluation of PCBs and PBDEs in cows’ milk is critical for identifying potential non-point sources and for establishing mitigation strategies for these persistent, organic pollutants in important food sources.

## Materials and Methods

### Sampling

Approximately 946 mL (one quart) of commercial bovine whole milk (WM) and fat free milk (FFM) samples from eight different brands were collected from high-delivery rate supermarkets in the Sacramento region between the months of August and September 2014. Each brand was selected based on whether the source and production of the milk originated from Californian farms. Of the eight brands, three were labelled as “organic”, while five brands were considered “conventional” milk. Geographic coordinates where milk samples were purchased are listed in S1 Table. Purchased milk was processed in the laboratory within one hour of purchase.

### Chemicals and reagents

Hexane, acetone, ethyl acetate, methylene chloride and isooctane (analytical grade), anhydrous magnesium sulfate and sodium chloride were purchased from Fisher Scientific (Pittsburgh, PA, USA). Ultrapure water (>18mΩ) was supplied by a Milli-Q system (Millipore, Billerica, MA, USA). The following analytical standards of PCBs were purchased from AccuStandard Inc. (New Haven, CT, USA): 3,3’-dichlorobiphenyl (PCB-11); 3,3,4,4’-tetrachlorobiphenyl (PCB-77); 2,2’,3,3’,6-pentachlorobiphenyl (PCB-84); 2,2’,3,4’,6-pentachlorobiphenyl (PCB-91); 2,2’,3,5’,6-pentachlorobiphenyl (PCB-95); 2,2’,4,5,5’-pentachlorobiphenyl (PCB-101); 2,3’,4,4’,5-pentachlorobiphenyl (PCB-118); 2,2’,3,3’,4,6-hexachlorobiphenyl (PCB-131); 2,2’,3,3’,4,6’-hexachlorobiphenyl (PCB-132); 2,2’,3,3’,5,6’-hexachlorobiphenyl (PCB-135); 2,2’,3,3’,6,6’-hexachlorobiphenyl (PCB-136); 2,2’,3,4,4’,5’-hexachlorobiphenyl (PCB138); 2,2’,3,4’,5’,6’,-hexachlorobiphenyl (PCB-149); 2,2’,4,4’,5,5’-hexachlorobiphenyl (PCB-153); 2,2’,3,3’,4,5,6’-heptachlorobiphenyl (PCB-174), 2,2’,3,3’,4,5’,6-heptachlorobiphenyl (PCB-175); 2,2’,3,3’,4,6,6’- heptachlorobiphenyl (PCB-176); 2,2’,3,4,4’,5,5’-heptachlorobiphenyl (PCB-180); 2,2’,3,3’,4,4’,5,6’-octachlorobiphenyl (PCB-196). The following analytical standards of PBDEs were purchased from AccuStandard Inc.: 2,2’,4-tribromodiphenyl ether (BDE-17); 2,4,4’-tribromodiphenyl ether (BDE-28); 2,2’,4,4’-tetrabromodiphenyl ether (BDE-47); 2,2’,4,5’-tetrabromodiphenyl ether (BDE-49); 2,3’,4,4’-tetrabromodiphenyl ether (BDE-66); 2,2’,3,4,4’-pentabromodiphenyl ether (BDE-85); 2,2’,3,5’,6-pentabromodiphenyl ether (BDE-95); 2,2’,4,4’,5-pentabromodiphenyl ether (BDE-99); 2,2’,4,5,5’-pentabromodiphenyl ether (BDE-100); 2,2’,4,4’,5,5’-hexabromodiphenyl ether (BDE-153); 2,2’,4,4’,5,6’-hexabromodiphenyl ether (BDE-154); 2,2’,3,4,4’,5’,6-heptabromodiphenyl ether (BDE-183). The ^13^C_12_-labeled surrogate internal standards ^13^C_12_-2,2’,3,4’,5-pentachlorobiphenyl (^13^C_12_-PCB-97) and ^13^C_12_-2,3’,4,4’,5-pentabromobiphenyl ether (^13^C_12_-BDE-118) were purchased from Cambridge Isotope Laboratories, Inc. (Andover, MA, USA). Mirex (Sigma-Aldrich Corp, St. Louis, MO, USA) was used as the secondary internal standard to assess instrument accuracy and bias.

### Sample extraction and clean-up

All samples were extracted by a modified version of the quick, easy, cheap, effective, rugged and safe (QuEChERS) method [42–44]. Each container of milk was brought to room temperature and homogenized by shaking before opening. A 5mL aliquot of milk was placed into a 50 ml polypropylene centrifuge tube and spiked with 10 μL of 100 ng/mL of internal standard. The sample was diluted with 5 mL of ultrapure water and extracted with 10 mL of hexane:acetone (1:1, v/v). Four grams of anhydrous magnesium sulfate and 1 g of sodium chloride were added to the mixture and the mixture was shaken for approximately 5 minutes using a platform shaker at 175 rpm. The sample was then centrifuged at 1606 xg for 5 minutes. The upper organic layer (~10mL) was transferred to a clean disposable glass tube and dried under a gentle stream of nitrogen at 40°C. Gel permeation chromatography (GPC) was used for clean-up. The extract was reconstituted in 5 mL of hexane:ethyl acetate (7:3, v/v), then filtered through a 0.45 *μ*m PTFE Acrodisc CR filter (Pall Corporation, CA, USA) and loaded onto the GPC (J2 Scientific, Columbia, MO, USA). The GPC column (700mm) was packed with 40 g of SX-3 biobeads (40–80 *μ*m) with 200–400 mesh size and a molecular weight (MW) exclusion limit of 14,000 (Bio-Rad Corporation, Hercules, CA, USA). With a flow rate of 5 ml/min, eluate was collected during 13.30–43.3 min in a 250 mL Erlenmeyer flask and evaporated under a gentle stream of nitrogen at 40°C. The residue was resolved in 100 *μ*L of isooctane prior to gas chromatography–tandem mass spectrometry (GC-MS/MS) analysis.

### GC-MS/MS analysis

The final sample extracts were analyzed for PCB and PBDE congeners using a Bruker Scion TQ triple quadruple mass spectrometer equipped with a Bruker 451 GC and CP 8400 autosampler (Bruker, Fremont, CA, USA). A BR-5MS column (15m×0.25 mm i.d×0.25 *μ*m) was used as the stationary phase. Helium was used as the carrier gas at a constant flow rate of 1.8 mL/min. The source and transfer line temperatures were set at 250°C and 280°C, respectively. The GC oven temperature gradient was as follows: 1) started at 90°C and held for 1 minute; 2) increased to 150°C at a rate of 50°C/min and held for 1 minute; 3) increased to 310°C at a rate of 8°C/min and held for 3 minutes. The MS/MS was operating in electron ionization (EI) positive mode at 70 eV. Two *μ*L of extract were injected by pulsed splitless mode (split ratio of 50:1, 50 psi, 0.2 min) at 250°C. The individual PCB and PBDE analytes were quantified using the sum of two multiple reactions monitoring (MRM) transitions and characterized by their abundance ratios [45].

### Quality control

To check the credibility of the data in the quantitative analyses, analytical validation of the method was performed. The factors considered in the validation included linearity, recovery, precision (relative standard deviation), accuracy, limit of detection (LOD) and limit of quantification (LOQ). Individual standard solutions of each PBDE (BDE-17, 28, 47, 49, 66, 85, 95, 99, 100, 153, 154, and 183) and PCB (PCB-11, 77, 84, 91, 95, 101, 118, 131, 132, 135, 136, 138, 149, 153, 174, 175, 176, 180 and 196) congener were combined to prepare a mixed standard solution. A calibration curve was constructed (for analysis of diluted whole milk (1:9 dilution with pure water, v/v) fortified by the addition of the mixed standard solution of PBDE and PCB congeners, at levels of 2, 4, 8, 20, 40, 80, and 200 pg/ml, respectively. Fortified standard curve samples were subjected to the same extraction and clean-up procedure as described above. To determine accuracy of the method, a recovery study was performed using whole milk or fat free milk fortified with PBDE and PCB analytical reference standards at three concentrations of 4, 20 and 80 pg/mL. The spiked milk samples were subjected to the same extraction and clean-up procedure as described above. Percentages of recoveries of each congener were determined in triplicate at each concentration. The average recovery (n = 9, 3 concentration levels × 3 biological replicates) and the relative standard deviation (RSD = standard deviation / average) are presented in S3 Table. The limit of detection (LOD) and limit of quantification (LOQ) were defined based on signal-to-noise ratio exceeding three and ten, respectively. Values found below the LOD were reported as “non-detected” (ND) and, for statistical purposes, were assigned a value of half the LOD.

### Lipids determination

Fat content of FFM and WM was determined by infrared (IR) spectroscopy following established standard operating procedures by the California Animal Health and Food Safety Laboratory, Milk Quality Section, San Bernardino, CA [46].

### Data analysis

The GC-MS/MS data was processed by Bruker Mass Spectrometry Working Station version 8.1 (Bruker, Fremont, CA, USA). The peak areas were used for quantification following an internal algorithm. Statistical analysis from the obtained data such as detection frequency, geometric mean (GM), geometric standard deviation (GSD), and median was done using Minitab version 17 software (Kivuto Solutions Inc. Ottawa, ON, Canada). Mann-Whitney Rank Sum Test was conducted to compare the differences between FFM and WM. Values of p < 0.05 were considered significant.

## Results and Discussion

We found detectable levels of PCBs and PBDEs in all eight brands of commercially available bovine milk samples purchased in California with LODs ranging 0.3–5.8 pg/mL and 0.2–3.5 pg/mL for PCBs and PBDEs, respectively as listed in Tables 1 and 2. The targeted PCB and PBDE congeners’ detection frequency was above 50%. The sample preparation method produced recoveries for PCBs and PBDEs of 66%-116.8% and 66%-117.6% from fat free milk (FFM), and 74.3%-120.3%and 74.8%-117.7% from whole milk (WM), respectively (S3 Table). The lipid corrected concentrations of PBDEs and PCBs in FFM and WM are presented in Table 3. Due to low fat content, the lipid adjusted concentrations of PBDE and PCBs in FFM were much higher than those in WM.

**Table 1:** The limit of detection (LOD), limit of quantification (LOQ), detection frequency (DF) and geometric mean (GM), geometric standard deviation (GSD) and median concentrations (pg/mL) of analyzed PCBs in eight brands of bovine milk samples analyzed in triplicate.

IUPAC Number	LOD	LOQ	DF	Fat Free Milk (n = 24)			Whole Milk (n = 23)			
	(pg/mL)	(pg/mL)	(%)	GM(pg/mL)	GSD	Median(pg/mL)	DF (%)	GM(pg/mL)	GSD	Median(pg/mL)
PCB-11	1.2	4.1	91.7	8.8	5.1	13.3	100.0	21.0	4.2	22.5
PCB-77	1.1	3.6	62.5	1.2	2.1	1.3	65.2	1.1	2.0	1.3
PCB-84	0.8	2.7	87.5	4.8	3.7	4.9	91.3	4.6	2.8	5.9
PCB-91	1.1	3.5	70.8	1.8	2.7	1.9	91.3	2.5	2.2	2.6
PCB-95	0.4	1.5	100.0	6.1	2.9	5.1	100.0	5.0	2.2	4.8
PCB-101	1.5	5.0	100.0	28.9	2.7	18.3	100.0	23.6	2.0	23.4
PCB-118	2.3	7.6	100.0	22.4	3.0	16.0	100.0	25.2	1.8	27.3
PCB-131	0.3	1.2	70.8	0.5	2.8	0.5	82.6	0.6	2.7	0.7
PCB-132	0.6	1.9	91.7	2.7	3.3	2.6	87.0	2.4	2.9	3.4
PCB-135	5.8	19.4	75.0	9.2	2.5	9.2	69.6	7.9	2.2	8.1
PCB-136	1.7	5.8	79.2	3.4	2.4	3.9	87.0	3.6	1.9	3.9
PCB-138	1.5	4.9	100.0	25.7	2.4	21.3	100.0	25.3	1.6	25.9
PCB-149	2.0	6.7	91.7	7.5	2.6	8.2	100.0	9.1	1.6	8.2
PCB-153	0.7	2.2	95.8	3.5	2.6	3.4	91.3	2.7	2.5	3.8
PCB-174	2.6	8.8	79.2	4.1	2.1	4.2	82.6	3.0	1.9	4.1
PCB-175	1.7	5.6	91.7	2.5	2.7	2.8	91.3	2.4	2.4	2.8
PCB-176	0.9	3.1	54.2	0.8	2.3	0.5	56.5	1.0	2.2	1.2
PCB-180	1.6	5.3	91.7	3.6	1.8	4.0	78.3	4.1	2.7	5.7
PCB-196	0.4	1.4	87.5	1.1	2.4	1.1	100.0	2.3	1.5	2.1
∑PCBs	NA	NA	NA	179.9	2.0	156.2	NA	189.9	1.5	172.4

NA: Not applicable

**Table 2:** The limit of detection (LOD), limit of quantification (LOQ), detection frequency (DF) and geometric mean (GM), geometric standard deviation (GSD) and median of analyzed PBDEs in eight brands of bovine milk samples analyzed in triplicate.

IUPAC Number	LOD	LOQ	DF	Fat Free Milk (n = 24)			Whole Milk (n = 23)			
	(pg/mL)	(pg/mL)	(%)	GM(pg/mL)	GSD	Median(pg/mL)	DF (%)	GM(pg/mL)	GSD	Median(pg/mL)
BDE-17	0.4	1.5	91.7	0.8	2.0	0.8	87.0	0.9	2.3	0.9
BDE-28	1.1	3.5	95.8	2.5	1.7	2.7	82.6	2.1	2.3	2.7
BDE-47	1.0	3.5	100.0	14.9	1.7	16.0	100.0	18.0	1.9	21.4
BDE-49	1.9	6.2	83.3	4.0	2.2	4.7	100.0	6.0	1.9	7.4
BDE-66	1.8	5.9	62.5	1.9	2.0	2.2	82.6	2.3	1.9	2.6
BDE-85	2.4	8.0	54.2	2.3	1.9	2.6	43.5	1.8	1.7	1.2
BDE-95	0.2	0.7	95.8	0.7	2.3	0.8	95.7	0.9	2.4	0.8
BDE-99	1.0	3.2	91.7	8.1	2.9	9.6	100.0	9.9	2.4	14.4
BDE-100	0.7	2.3	100.0	3.9	1.6	3.9	100.0	5.0	1.5	4.4
BDE-153	0.8	2.6	87.5	1.8	2.3	2.1	100.0	2.6	1.7	2.5
BDE-154	1.2	4.0	87.5	3.0	2.5	3.1	87.0	2.9	2.5	3.1
BDE-183	3.5	11.5	50.0	3.4	2.2	2.7	91.3	5.5	2.0	6.4
∑PBDEs	NA	NA	NA	55.3	1.5	58.5	NA	65.7	1.5	76.5

NA: Not applicable

**Table 3:** The geometric mean (GM), geometric standard deviation (GSD) and median of analyzed PCBs and PBDE concentrations corrected by milk fat (ng/g lipid) in eight brands of bovine milk samples analyzed in triplicate.

IUPAC Number	Fat Free Milk (n = 24)			Whole Milk (n = 23)		
	GM	GSD	Median	GM	GSD	Median
	(ng/g lipid)		(ng/g lipid)	(ng/g lipid)		(ng/g lipid)
PCB-11	6.08	6.04	11.39	0.60	4.30	0.63
PCB-77	0.81	2.95	0.83	0.03	1.96	0.03
PCB-84	3.32	6.02	4.51	0.13	2.79	0.17
PCB-91	1.22	3.79	1.21	0.07	2.11	0.08
PCB-95	4.26	4.37	3.71	0.14	2.25	0.13
PCB-101	20.05	4.72	18.85	0.67	1.96	0.64
PCB-118	15.54	5.60	18.77	0.72	1.76	0.74
PCB-131	0.37	3.45	0.52	0.02	2.70	0.02
PCB-132	1.90	5.32	2.49	0.07	2.83	0.10
PCB-135	6.39	3.83	7.60	0.22	2.27	0.22
PCB-136	2.32	3.75	2.43	0.10	1.93	0.12
PCB-138	17.81	4.57	19.20	0.72	1.60	0.72
PCB-149	5.18	4.33	6.99	0.26	1.63	0.23
PCB-153	2.40	4.61	2.83	0.08	2.54	0.10
PCB-174	2.83	3.14	4.02	0.08	1.92	0.12
PCB-175	1.72	3.51	1.83	0.07	2.30	0.08
PCB-176	0.58	2.72	0.49	0.03	2.24	0.03
PCB-180	2.48	2.70	2.95	0.12	2.62	0.16
PCB-196	0.74	3.43	0.95	0.06	1.49	0.06
∑PCBs	124.7	3.66	142.85	5.39	1.52	4.86
BDE-17	0.58	2.79	0.55	0.03	2.36	0.02
BDE-28	1.75	2.65	2.45	0.06	2.39	0.08
BDE-47	10.35	2.48	11.97	0.51	1.92	0.59
BDE-49	2.76	3.21	3.60	0.17	1.93	0.20
BDE-66	1.35	3.20	1.85	0.06	1.87	0.07
BDE-85	1.59	2.74	1.55	0.05	1.66	0.04
BDE-95	0.51	3.18	0.69	0.03	2.39	0.02
BDE-99	5.61	3.50	7.64	0.28	2.52	0.41
BDE-100	2.71	2.94	3.88	0.14	1.51	0.12
BDE-153	1.25	3.22	1.44	0.07	1.69	0.07
BDE-154	2.05	3.21	2.19	0.08	2.58	0.09
BDE-183	2.38	2.50	2.26	0.16	1.94	0.18
∑PBDEs	38.4	2.43	45.80	1.86	1.55	1.96

**Fig 1:**
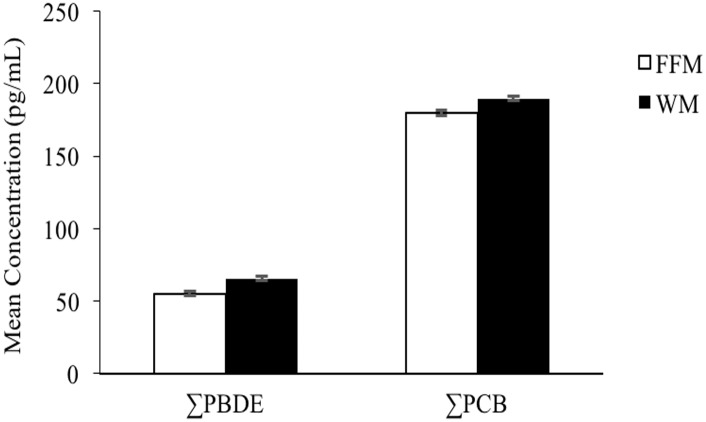
The concentrations of ∑PBDEs and ∑PCBs in fat free milk and whole milk. Data shown are the geometric mean (pg/mL) with geometric standard deviation, of eight brands of milk samples analyzed in triplicate (fat free milk n = 24, whole milk n = 23).

Overall, total PCB concentrations (pg/mL) were higher than total PBDE concentrations in both fat free milk and whole milk. PCB-138 (GM: 25.7 and 25.3 pg/mL for FFM and WM), PCB-101 (GM: 28.9 and 23.6 pg/mL for FFM and WM) and PCB-118 (GM: 22.4 and 25.2 pg/mL for FFM and WM) were the top three contributors among all analyzed PCBs. BDE-47 (GM: 14.9 and 18.0 pg/mL for FFM and WM), BDE-99 (median: 8.1 and 9.9 pg/mL for FFM and WM) and BDE-49 (GM: 4.0 and 6.0 pg/mL for FFM and WM) were the top three dominant PBDE congeners among all analyzed PBDEs (Table 2). ∑PBDEs and ∑PCBs were slightly higher in WM (∑PBDEs and ∑PCBs GM: 65.7 and 189.9 pg/mL, respectively) compared to FFM (∑PBDEs and ∑PCBs GM: 55.3 and 179.9 pg/mL, respectively). The differences were not statistically significant (Fig 1).

**Fig 2:**
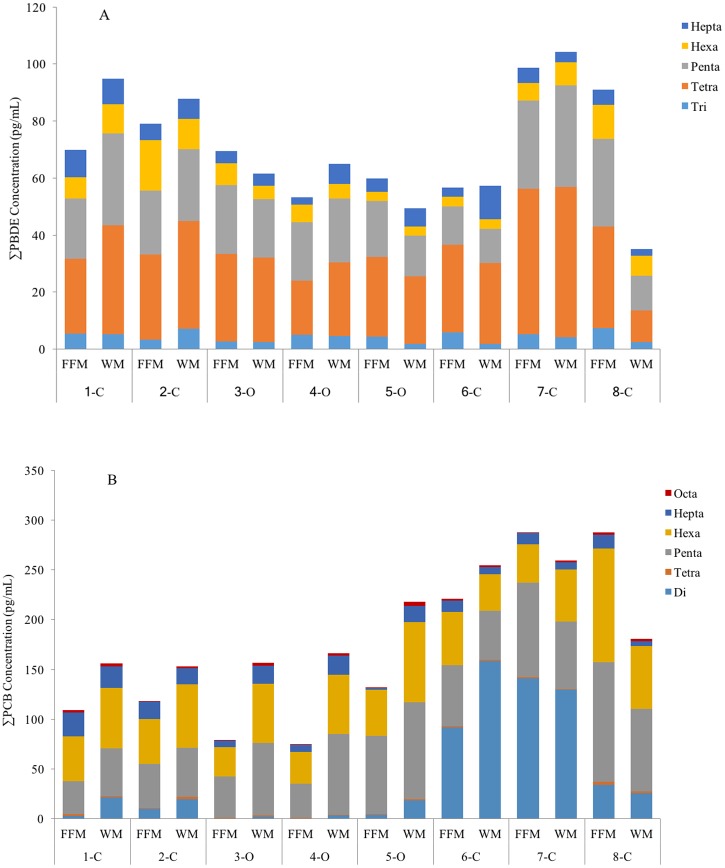
The concentrations of ∑PBDEs and ∑PCBs with the relative amount of chlorination and bromination. (A) ∑PBDEs (Tri: BDE-17 and -28, Tetra: BDE-47, -49 and -66; Penta: BDE-85, -95, -99 and -100; Hexa: BDE-153 and -154; Hepta: BDE-183). (B) ∑PCBs (Di: PCB-11; Tetra: PCB-77; Penta: PCB-84, -91, -95, -101 and -118; Hexa: PCB-131, -132, -135, -136, -138, -149 and -153; Hepta: PCB-174, -175, -176 and -180; Octa: PCB-196). Number 1 to 8 represent eight different brands of commercial milk. ‘C’ indicates conventional milk, while ‘O’ indicates organic milk. Data shown are the geometric mean (pg/mL).

**Fig 3:**
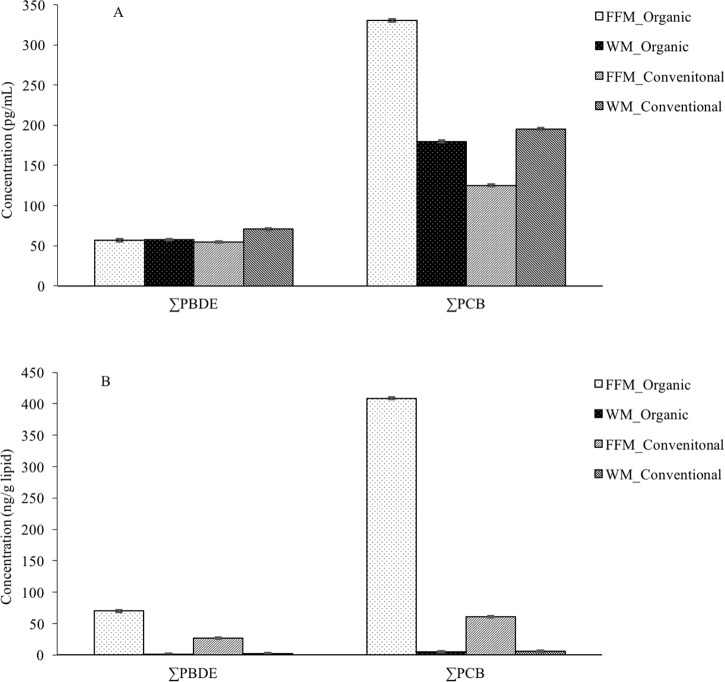
∑PBDE and ∑PCB concentrations in organic and conventional milk. (A) Concentrations based on milk volume (pg/mL). (B) Concentrations based on milk lipid (ng/g lipid). Data shown are the geometric mean with geometric standard deviation of each brand of milk sample analyzed in triplicate (n = 9 for organic brands FFM, n = 8 for organic brands WM, and n = 15 for conventional brands FFM and WM).

The concentrations of ∑PCBs and ∑PBDEs in individual brands varied between FFM and WM (Fig 2). For most brands, ∑PBDEs and ∑PCBs were higher in WM than FFM. However, the ∑PBDEs of brand #3, 5, 8 were higher in FFM (GM of brands# 3, 5, 8: 69.4, 59.8 and 90.9 pg/mL) compared to WM (GM of brand # 3, 5, 8: 61.7, 49.4, and 35.1 pg/mL). In addition, ∑PCBs of brand # 7, 8 were higher in FFM (GM of brand # 7, 8: 287.8 and 287.9 pg/mL) compared to WM (GM of brand # 7, 8: 259.6 and 180.7 pg/mL). None of the differences between FFM and WM in the eight brands were statistically significant. As shown in Fig 3, the ∑PBDEs in organic FFM (GM: 56.6 pg/mL) was slightly lower compared to organic WM (GM: 57.5 pg/mL). In contrast, ∑PCBs in organic FFM (GM: 330.2 pg/mL) was higher compared to that in organic WM (GM: 179.8 pg/mL). For conventional milk (brands #1, 2, 6, 7, 8), ∑PCBs and ∑PBDEs were lower in FFM (GM: 124.9 pg/mL and 54.6 pg/mL for PCBs and PBDEs, respectively) compared to WM (GM: 195.4 pg/mL and 70.5 pg/mL for PCBs and PBDEs).

### PCB concentrations compared to the FDA tolerance and other studies

The U.S. Food and Drug Administration (FDA) tolerance for PCBs in fluid milk is 1.5 μg/g based on milk fat (http://www.accessdata.fda.gov/scripts/cdrh/cfdocs/cfcfr/CFRSearch.cfm?fr=109.30). Because PCBs and PBDEs are lipophilic, we report PCB and PBDE concentrations based on milk volume (mL) basis (pg/mL) and fat content basis (ng/g lipid). The geometric mean concentrations of lipid-adjusted ∑PCBs in WM and FFM samples were 5.4 and 124.7 ng/g respectively, which are both several orders of magnitude below the U.S. FDA tolerance of 1.5 μg/g milk fat. However, it is important to note that our data is comprised of only 19 PCB congeners. The concentrations of ∑PCBs found in WM (GM: 189.9 pg/mL) and FFM (GM: 179.9 pg/mL) in our samples were higher compared to data generated from a 2000/2001 U.S. survey which reported 14.1 pg/mL as the sum of PCB-77, -118, -105, -126, -156, -157, and -169, identifying PCB-118 and -105 as the major contributors [34]. The study by Schaum et al. evaluated geographical differences and identified that ∑PCBs tend to be lower in the West Central region compared to the Far West and Mid-Atlantic regions [34]. Our samples were collected from the Far West region when applying the geographic division approach used in that study. When comparing the geometric means of PCB-77 and -118 of 1.1 pg/mL and 25.2 pg/mL in WM and 1.2 pg/mL and 22.4 pg/mL in FFM to the 2003 national average for unprocessed milk collected from contributing dairies of 0.0711 pg/mL (PCB-77) and 9.64 pg/mL (PCB-118), our results are higher. The levels and % contribution of individual congeners to total PCBs vary between studies and countries. In our study, PCB-138 was found in highest concentrations (GM: 25.3 pg/mL), followed by -118 (GM: 25.2 pg/mL), -101 (GM: 23.6 pg/mL), -11 (GM: 21.0 pg/mL), and -149 (GM: 9.1 pg/mL) in WM. This congener pattern differs from that seen in other studies from European countries, including Finland, the UK, and Spain [47–49], African countries (Ghana and South Africa) [50, 51], and Mexico. In those studies, PCB-153 and -138 were the most dominant congeners. There are known seasonal effects on PCB levels in bovine milk because of a washing effect subsequent to rainy conditions on PCB levels in soil and forage [52]. In addition, feeding practices for dairy cows vary widely between countries and regions with possible impact on different level and congener pattern exposure to PCBs. Another important factor that influences levels and congener patterns of PCBs in milk is days in lactation [53].

Interestingly PCB-11, known to be absent in the production of commercial PCB products, was detected above the LOQ in all milk samples and accounted for 5% and 11% of ∑PCBs in FFM and WM (according to the GM), respectively. PCB-11 is identified as a marker of non-legacy PCB contamination, and has been traced back to paint manufacturing facilities. Specifically, PCB-11 is produced in the production of diarylide yellow pigments [54, 55], and not considered a metabolite of historical PCB mixtures. It has been found in polar region air samples, urban and rural environments in the US, and in Great Lakes Sediment [54, 56–58]. Dairy feeds can be contaminated through sewage sludge, coatings of silos, air-soil deposition from industrial emissions, and run-off from wastewater treatment plants. In addition, husbandry of cattle can expose them to painted fences, barns and hutches, and corral pipes and subsequent exposure to paints and pigments are potential sources for exposure to non-legacy PCBs, such as PCB-11. Our findings highlight the recent recognition that unintentional by-product PCBs need to be monitored more rigorously to better understand their contribution to overall PCB exposure in humans, animals, and the environment.

### PBDE concentrations compared to other studies

There are currently no tolerances established for PBDEs in fluid milk in the US [59]. Surprisingly, total PBDEs in bovine fluid milk have increased in various countries. In the U.S., ∑PBDEs in commercial cows’ milk increased by 28.8% between 2003 and 2009 [60, 61]. Similarly, time trend studies from Sweden showed a 45% increase in levels of ∑PBDEs between 1999 and 2005 [59, 62]. Specifically, time trend studies from Sweden showed a 62.5% increase in levels of BDE-47 between 1999 (0.005 ng/g wet weight) and 2005 (0.008 ng/g wet weight) [59, 62]. In the U.S., BDE-99 and BDE-100 in bovine whole milk collected in Dallas, Texas (U.S.) increased by 22% and 100% at 2009 compared to data from 2003 [60, 61]. BDE-47 was not detected in 2003 but identified as a major PDBE contributor with 2.57 pg/g wet weight in 2009. In our study, WM contained 18.0 pg/ml (GM) of BDE-47, which was identified as the dominant congener amongst the 12 congeners determined. The concentrations of BDE-99 (GM: 9.9 pg/mL) and BDE-100 (GM: 5.0 pg/mL) found in our WM samples were lower than the average concentrations of BDE-99 and BDE-100 found in milk samples collected in Dallas, Texas (US) in 2003 (1.58 ng/g wet weight, 0.23 ng/g wet weight) [60] and 2009 (1.93 ng/g wet weight and 0.46 ng/g wet weight) [61].

PBDE congener patterns observed in our milk study relate with that of commercial PBDE mixtures in North America and Europe [63]. BDE-47 and BDE-99 were the most abundant congeners in our study. In the U.S., DE-71 is considered the primary commercial PBDE product with mass production between 1976 and 2004 [63, 64]. DE-71 is a penta-BDE mixture, comprised mainly of BDE-47 and BDE-99 (>70%), with smaller contributions from BDE-100, BDE-153, BDE-154 and trace levels of other lower brominated congeners (<2%) [64, 65]. BDE-47 and BDE-99 have been identified as the major contributing congeners in milk in various regions of the world, including Finland [47], Italy [66], Spain [67], and Ghana [51]. Interestingly, in our milk samples BDE-49 contributed to a much larger degree to ∑PBDEs (7.2% in FFM and 9.1% in WM based on GM), compared to its proportion in DE-71 (< 1%) [68]. This finding may be a result of metabolism of higher brominated congeners. Animal studies have shown that highly brominated PBDE congeners, such as deca-BDEs, can be de-brominated and metabolized to lower brominated congeners [24, 25] leading to increased levels of lower brominated PBDE congeners *in vivo*. *In vitro* studies have shown differences in biotransformation between BDE-47 and BDE-99 using human liver microsomes [22, 69]. While there are no data on metabolism of PBDEs in bovine, BDE-99 has been shown to be de-brominated to BDE-49 in Chinook Salmon[27]. BDE-99 was also shown to be more extensively metabolized than BDE-47 in rats [28]. It is well established that biotransformation pathways are influenced by many biological factors, including species, age, hormone status, pregnancy, preexisting health conditions, and genetic polymorphisms [29].

We considered the potential sources of contamination of PBDEs and PCBs in commercial bovine milk. We provided information on the geographic location where milk was purchased in S1 Table. It is important to note that information as to the location of the dairy processor does not allow for information down to the level of location of contributing farms towards an individual lot of produced milk. Based on the fact that all of our milk samples are from dairy processors in the Northern Region of California, we can merely speculate as to the possible sources of contamination. Diet could be an exposure source for PBDE and PCBs in dairy cows. Dairy cows are usually kept in constant environments being fed a total mixed ration consisting of specific commodity components. The total mixed ration typically contains corn and or corn silage, a feed component documented to contain PCBs and PBDEs [70–72]. There are studies reporting PCB levels in fresh grass from the United Kingdom and corn from Italy [72, 73]. While the locations for these feed ingredients differed, corn samples had 10–1000 times the PCB content measured in grass. This may be due to the fact that corn contains a large amount of fatty acids [74] which would allow easier partitioning of these lipophilic pollutants into the roots or leaves. Various forms of corn that are used in feed have lipid percentages ranging from 3.8–10% while those among different grasses range from 2.0–4.1% [75]. Based on these data, rations including corn, such as typical dairy cow rations, may contain higher levels of PCBs and PBDEs than animals grazing pastures. Thus, dairy cattle may be exposed to higher levels of these contaminants through their diets.

More specific to the location of the dairy processors in our study, we speculate that the use of flame retardants may play a role in the detected PBDE contaminant levels. Many of the Northern California counties are located in areas that are at high risk for wildfires, or where wildfires have occurred in California in recent years (http://frap.fire.ca.gov/webdata/maps/statewide/fhszs_map.pdf). It is likely that flame retardants were used in these regions due to the fire risk, and also due to the high level of firefighting activities ongoing in the area. This conclusion is supported by multiple pieces of evidence. One being the fact that PBDEs are in fact present in firefighter equipment [76]. Second, California firefighters themselves that have been studied and results from two different populations show that due to their activities their sera contain levels of PBDEs far above those found in the general California population [77, 78]. Third, the dust from a total of 20 fire stations in Southern California contained higher levels of PBDEs compared to dust in residential homes [79]. A common factor among these mentioned studies are the PBDEs that are most prominent or make up the dominant portion namely BDE-47, -99, -153, -154, and -209. This directly ties back to our dataset with the PBDE congeners that are present in our milk samples shown in Table 3. California mainly used penta-BDE mixtures before they were banned, and the major components of these mixtures were BDE-47, -99, -153, and -154 [80] adding further support for the increased use of flame retardants in Northern California contributing to the PBDE content in bovine milk made in Northern California. The major PBDE congeners of past flame retardant mixtures along with the prominent ones present in firefighter’s clothing and serum and the dust of fire stations are PBDE congeners that are higher in serum from the Northern region of California.

While the industrial processes are considered less likely to result in milk contamination, materials used during processing and storage must be considered. Raw milk is generally stored in stainless steel tanks until processing. Processed milk may be stored in containers made of plastic. Based on a study by Webb et al. [81] plastics can degrade in the environment by four mechanisms: photodegradation, thermooxidative degradation, hydrolytic degradation and biodegradation by microorganisms. None of these degradation processes are likely to occur during storage and processing of milk. Thus, while contamination during processing cannot be completely ruled out, it is an unlikely source of PCB or PBDE contamination.

Although this study fills important data gaps, some limitations are worth consideration. Each group of milk samples consisted of only eight different brands and samples were only collected in the central valley of California at one time point. Differences in PCB and PBDE congener patterns between our data and those reported from other studies may be a result of different congener mixtures being used, especially when comparing results to other countries. In addition, differences in husbandry and feeding practices, regional distinctions affecting contaminant levels such as run-off from wastewater treatment plants, industrial emissions, or air-soil deposition differences, may contribute to the observed differences. Despite these limitations, the major focus of this research is the assessment of PBDEs and PCBs in dietary milk ready for human consumption in California compared to other national and international studies. Clearly there is much more work to be done in this area, and we expect that further work will uncover additional factors that contribute to the occurrences and differences in PBDE and PCB levels in animal-derived foods.

## Conclusions

In summary, contamination of bovine milk with PCBs and PBDEs is not unexpected and was confirmed in our study. The ∑PCBs in bovine milk samples were several orders of magnitude below the U.S. FDA tolerance for milk fat. Our findings provide further evidence for the importance of assessing non-legacy PCBs, such as PCB-11, in dietary sources. ∑PBDEs determined in our milk samples suggest the dynamic profile of PBDE concentrations based on geographic regions and calendar time. Finally, regular screening of dairy products is essential to assess the occurrence of contributions from dietary sources for environmental contaminant exposure.

## Supporting Information

S1 Table Milk purchase information. (XLSX)

S2 Table Raw data (PCB and PBDE concentrations in pg/ml) for all analyzed samples (in triplicates). (XLSX)

S3 Table Average recovery and the relative standard deviation for each congener (n = 9, 3 concentration levels × 3 biological replicates). (XLSX)

## References

[1] Sjödin A, Jones RS, Focant J-F, Lapeza C, Wang RY, McGahee EE, et al Retrospective time-trend study of polybrominated diphenyl ether and polybrominated and polychlorinated biphenyl levels in human serum from the United States. Environ Health Perspect. 2004;112(6):654–8. 15121506 10.1289/ehp.112-1241957 PMC1241957

[2] Costa LG, Giordano G, Tagliaferri S, Caglieri A. Polybrominated diphenyl ether (PBDE) flame retardants: environmental contamination, human body burden and potentialadverse health effects. Acta Bio Medica Atenei Parmensis. 2009;79(3):172–83. 19260376

[3] Talsness CE, Andrade AJ, Kuriyama SN, Taylor JA, vom Saal FS. Components of plastic: experimental studies in animals and relevance for human health. Philos Trans R Soc Lond B Biol Sci. 2009;364(1526):2079–96. 10.1098/rstb.2008.0281 19528057 PMC2873015

[4] ATSDR. Toxicological profile for polybrominated biphenyls and polybrominated diphenyl ethers. Atlanta, GA: Agency for Toxic Substances and Disease Registry 2004. 38412208

[5] Hale RC, La Guardia MJ, Harvey E, Mainor TM. Potential role of fire retardant-treated polyurethane foam as a source of brominated diphenyl ethers to the US environment. Chemosphere. 2002;46(5):729–35. 11999796 10.1016/s0045-6535(01)00237-5

[6] Dodson RE, Perovich LJ, Covaci A, Van den Eede N, Ionas AC, Dirtu AC, et al After the PBDE phase-out: A broad suite of flame retardants in repeat house dust samples from California. Environ Sci Technol. 2012;46(24):13056–66. 10.1021/es303879n 23185960 PMC3525011

[7] Information CL. AB-302 Pupil services: lactation accommodations.(2015–2016) [cited 2015]. http://leginfo.legislature.ca.gov/faces/billNavClient.xhtml?bill_id=201520160AB302.

[8] Pessah IN, Cherednichenko G, Lein PJ. Minding the calcium store: Ryanodine receptor activation as a convergent mechanism of PCB toxicity. Pharmacol Ther. 2010;125(2):260–85. Epub 2009/11/26. 10.1016/j.pharmthera.2009.10.009 19931307 PMC2823855

[9] Safe S. Molecular biology of the Ah receptor and its role in carcinogenesis. Toxicol Lett. 2001;120(1–3):1–7. 11323156 10.1016/s0378-4274(01)00301-0

[10] Safe S, Astroff B, Harris M, Zacharewski T, Dickerson R, Romkes M, et al 2,3,7,8-Tetrachlorodibenzo-p-dioxin (TCDD) and related compounds as antioestrogens: characterization and mechanism of action. Pharmacol Toxicol. 1991;69(6):400–9. Epub 1991/12/01. 1766914 10.1111/j.1600-0773.1991.tb01321.x

[11] Safe SH. Polychlorinated biphenyls (PCBs): environmental impact, biochemical and toxic responses, and implications for risk assessment. Crit Rev Toxicol. 1994;24(2):87–149. 10.3109/10408449409049308 8037844

[12] Bae J, Peters-Golden M, Loch-Caruso R. Stimulation of pregnant rat uterine contraction by the polychlorinated biphenyl (PCB) mixture aroclor 1242 may be mediated by arachidonic acid release through activation of phospholipase A(2) enzymes. J Pharmacol Exp Ther. 1999;289(2):1112–20. 10215694

[13] Khan MA, Lichtensteiger CA, Faroon O, Mumtaz M, Schaeffer DJ, Hansen LG. The hypothalamo-pituitary-thyroid (HPT) axis: A target of nonpersistent ortho-substituted PCB congeners. Toxicol Sci. 2002;65(1):52–61. 11752685 10.1093/toxsci/65.1.52

[14] Fischer LJ, Wagner MA, Madhukar BV. Potential involvement of calcium, CaM kinase II, and MAP kinases in PCB-stimulated insulin release from RINm5F cells. Toxicol Appl Pharmacol. 1999;159(3):194–203. 10.1006/taap.1999.8728 10486306

[15] Li MH, Hansen LG. Enzyme induction and acute endocrine effects in prepubertal female rats receiving environmental PCB/PCDF/PCDD mixtures. Environ Health Perspect. 1996;104(7):712–22. 8841756 10.1289/ehp.96104712 PMC1469418

[16] Wong PW, Pessah IN. Ortho-substituted polychlorinated biphenyls alter calcium regulation by a ryanodine receptor-mediated mechanism: structural specificity toward skeletal- and cardiac-type microsomal calcium release channels. Mol Pharmacol. 1996;49(4):740–51. Epub 1996/04/01. 8609904

[17] Wong PW, Brackney WR, Pessah IN. Ortho-substituted polychlorinated biphenyls alter microsomal calcium transport by direct interaction with ryanodine receptors of mammalian brain. J Biol Chem. 1997;272(24):15145–53. Epub 1997/06/13. 9182535 10.1074/jbc.272.24.15145

[18] Shain W, Bush B, Seegal R. Neurotoxicity of polychlorinated biphenyls: structure-activity relationship of individual congeners. Toxicol Appl Pharmacol. 1991;111(1):33–42. 1949034 10.1016/0041-008x(91)90131-w

[19] Toxicological profile for polybrominated biphenyls and polybrominated diphenyl ethers (PBBs and PBDEs). [Atlanta, GA]:: U.S. Dept. of Health and Human Services, Public Health Service, Agency for Toxic Substances and Disease Registry; 2004.

[20] Darnerud PO. Toxic effects of brominated flame retardants in man and in wildlife. Environ Int. 2003;29(6):841–53. 10.1016/S0160-4120(03)00107-7 12850100

[21] Feo ML, Gross MS, McGarrigle BP, Eljarrat E, Barcelo D, Aga DS, et al Biotransformation of BDE-47 to potentially toxic metabolites is predominantly mediated by human CYP2B6. Environ Health Perspect. 2013;121(4):440–6, 6e1–7. Epub 2012/12/20. 10.1289/ehp.1205446 23249762 PMC3620761

[22] Erratico CA, Szeitz A, Bandiera SM. Oxidative Metabolism of BDE-99 by Human Liver Microsomes: Predominant Role of CYP2B6. Toxicol Sci. 2012;129(2):280–92. 10.1093/toxsci/kfs215 22738989

[23] EPA. Toxicological Review of Decabromodiphenyl ether (BDE-209)2008. www.epa.gov/iris.

[24] Sandholm A, Emanuelsson BM, Wehler EK. Bioavailability and half-life of decabromodiphenyl ether (BDE-209) in rat. Xenobiotica. 2003;33(11):1149–58. Epub 2003/12/09. 10.1080/00498250310001609156 14660178

[25] Huwe JK, Smith DJ. Accumulation, whole-body depletion, and debromination of decabromodiphenyl ether in male Sprague-Dawley rats following dietary exposure. Environ Sci Technol. 2007;41(7):2371–7. 17438789 10.1021/es061954d

[26] Thuresson K, Hoglund P, Hagmar L, Sjodin A, Bergman A, Jakobsson K. Apparent half-lives of hepta- to decabrominated diphenyl ethers in human serum as determined in occupationally exposed workers. Environ Health Perspect. 2006;114(2):176–81. 10.1289/ehp.8350 16451851 PMC1367828

[27] Browne EP, Stapleton HM, Kelly SM, Tilton SC, Gallagher EP. In vitro hepatic metabolism of 2,2 ',4,4 ',5-pentabromodiphenyl ether (BDE 99) in Chinook Salmon (Onchorhynchus tshawytscha). Aquat Toxicol. 2009;92(4):281–7. 10.1016/j.aquatox.2009.02.017 19346012 PMC2739728

[28] Chen LJ, Lebetkin EH, Sanders JM, Burka LT. Metabolism and disposition of 2,2',4,4',5-pentabromodiphenyl ether (BDE99) following a single or repeated administration to rats or mice. Xenobiotica. 2006;36(6):515–34. Epub 2006/06/14. 10.1080/00498250600674477 16769647

[29] Factors that Influence Drug Biotransformation In: Ionescu C, Caira MR, editors. Drug Metabolism: Current Concepts. Dordrecht: Springer Netherlands; 2005 p. 243–68.

[30] Durand B, Dufour B, Fraisse D, Defour S, Duhem K, Le-Barillec K. Levels of PCDDs, PCDFs and dioxin-like PCBs in raw cow's milk collected in France in 2006. Chemosphere. 2008;70(4):689–93. 10.1016/j.chemosphere.2007.06.057 17707881

[31] Ruoff U, Karl H, Walte HG. Dioxins, dioxin-like PCBs and non-dioxin-like PCBs in dairy products on the German market and the temporal tendency in Schleswig-Holstein. J Verbrauch Lebensm. 2012;7(1):11–7.

[32] Ucar Y, Traag W, Immerzeel J, Kraats C, van der Lee M, Hoogenboom R, et al Levels of PCDD/Fs, PCBs and PBDEs in butter from Turkey and estimated dietary intake from dairy products. Food Addit Contam B. 2011;4(2):141–51. 10.1080/19440049.2011.576435 21557123

[33] Brambilla G, Abate V, De Filippis SP, Fulgenzi AR, Iamiceli AL, Mazzette A, et al Polychlorodibenzodioxin and -furan (PCDD and PCDF) and dioxin-likepolychlorobiphenyl (DL-PCB) congener levels in milk of grazing sheep as indicators of the environmental quality of rural areas. J Agric Food Chem. 2011;59(15):8513–7. 10.1021/jf2010673 21699241

[34] Schaum J, Schuda L, Wu C, Sears R, Ferrario J, Andrews K. A national survey of persistent, bioaccumulative, and toxic (PBT) pollutants in the United States milk supply. J Expo Anal Environ Epidemiol. 2003;13(3):177–86. 10.1038/sj.jea.7500269 12743612

[35] Bozlaker A, Odabasi M, Muezzinoglu A. Dry deposition and soil-air gas exchange of polychlorinated biphenyls (PCBs) in an industrial area. Environ Pollut. 2008;156(3):784–93. 10.1016/j.envpol.2008.06.008 18640753

[36] Clarke BO, Porter NA, Marriott PJ, Blackbeard JR. Investigating the levels and trends of organochlorine pesticides and polychlorinated biphenyl in sewage sludge. Environ Int. 2010;36(4):323–9. 10.1016/j.envint.2010.01.004 20171737

[37] Wang Y, Zhang Q, Lv J, Li A, Liu H, Li G, et al Polybrominated diphenyl ethers and organochlorine pesticides in sewage sludge of wastewater treatment plants in China. Chemosphere. 2007;68(9):1683–91. 10.1016/j.chemosphere.2007.03.060 17509654

[38] Tremolada P, Guazzoni N, Parolini M, Rossaro B, Bignazzi MM, Binelli A. Predicting PCB concentrations in cow milk: validation of a fugacity model in high-mountain pasture conditions. Sci Total Environ. 2014;487:471–80. 10.1016/j.scitotenv.2014.04.042 24802270

[39] Chobtang J, de Boer IJ, Hoogenboom RL, Haasnoot W, Kijlstra A, Meerburg BG. The need and potential of biosensors to detect dioxins and dioxin-like polychlorinated biphenyls along the milk, eggs and meat food chain. Sensors (Basel). 2011;11(12):11692–716. 22247688 10.3390/s111211692 PMC3252005

[40] Sweetman AJ, Thomas GO, Jones KC. Modelling the fate and behaviour of lipophilic organic contaminants in lactating dairy cows. Environ Pollut. 1999;104(2):261–70.

[41] EFSA. Results of the monitoring of non dioxin-like PCBs in food and feed. EFSA Journal. 2010;8(7):35.

[42] Sapozhnikova Y, Simons T, Lehotay SJ. Evaluation of a ast and simple sample preparation method for polybrominated diphenyl ether (PBDE) flame retardants and dichlorodiphenyltrichloroethane (DDT) pesticides in fish for analysis by ELISA compared with GC-MS/MS. J Agric Food Chem. 2015;63(18):4429–34. Epub 2015/02/04. 10.1021/jf505651g 25644932

[43] Sapozhnikova Y, Lehotay SJ. Multi-class, multi-residue analysis of pesticides, polychlorinated biphenyls, polycyclic aromatic hydrocarbons, polybrominated diphenyl ethers and novel flame retardants in fish using fast, low-pressure gas chromatography-tandem mass spectrometry. Anal Chim Acta. 2013;758:80–92. Epub 2012/12/19. 10.1016/j.aca.2012.10.034 23245899

[44] Zacs D, Rjabova J, Viksna A, Bartkevics V. Method development for the simultaneous determination of polybrominated, polychlorinated, mixed polybrominated/chlorinated dibenzo-p-dioxins and dibenzofurans, polychlorinated biphenyls and polybrominated diphenyl ethers in fish. Chemosphere. 2015;118:72–80. Epub 2014/07/12. 10.1016/j.chemosphere.2014.06.032 25014661

[45] Lin YP, Pessah IN, Puschner B. Simultaneous determination of polybrominated diphenyl ethers and polychlorinated biphenyls by gas chromatography-tandem mass spectrometry in human serum and plasma. Talanta. 2013;113:41–8. Epub 2013/05/28. 10.1016/j.talanta.2013.04.001 23708622 PMC3698050

[46] Wehr M, Frank JF. Standard Methods for the Examination of Dairy Products: American Public Health Association; 2004.

[47] Kiviranta H, Ovaskainen ML, Vartiainen T. Market basket study on dietary intake of PCDD/Fs, PCBs, and PBDEs in Finland. Environ Int. 2004;30(7):923–32. 10.1016/j.envint.2004.03.002 15196840

[48] Sewart A, Jones KC. A survey of PCB congeners in UK cows' milk. Chemosphere. 1996;32(12): 2481–92. 8653383 10.1016/0045-6535(96)00141-5

[49] Luzardo OP, Almeida-Gonzalez M, Henriquez-Hernandez LA, Zumbado M, Alvarez-Leon EE, Boada LD. Polychlorobiphenyls and organochlorine pesticides in conventional and organic brands of milk: occurrence and dietary intake in the population of the Canary Islands (Spain). Chemosphere. 2012;88(3):307–15. 10.1016/j.chemosphere.2012.03.002 22472097

[50] Batterman S, Chernyak S, Gouden Y, Hayes J, Robins T, Chetty S. PCBs in air, soil and milk in industrialized and urban areas of KwaZulu-Natal, South Africa. Environ Pollut. 2009;157(2):654–63. 10.1016/j.envpol.2008.08.015 18838199 PMC4365072

[51] Asante KA, Sudaryanto A, Devanathan G, Bello M, Takakashi S, Isobe T, et al. Polybrominated diphenyl ethers and polychlorinated biphenyls in cow milk samples from Ghana. Interdiscipl Studies Environ Chem. 2010;Environmental Specimen Bank:191–8.

[52] Perez JJ, Leon SV, Gutierrez R, Lopez Y, Faure R, Escobar A. Polychlorinated biphenyls (PCBs) residues in milk from an agroindustrial zone of Tuxpan, Veracruz, Mexico. Chemosphere. 2012;89(4):404–8. 10.1016/j.chemosphere.2012.05.055 22739542

[53] Thomas GO, Sweetman AJ, Jones KC. Metabolism and body-burden of PCBs in lactating dairy cows. Chemosphere. 1999;39(9):1533–44. 10481252 10.1016/s0045-6535(99)00050-8

[54] Rodenburg LA, Guo J, Du S, Cavallo GJ. Evidence for unique and ubiquitous environmental sources of 3,3'-dichlorobiphenyl (PCB 11). Environ Sci Technol. 2010;44(8):2816–21. 10.1021/es901155h 20384375

[55] Shang H, Li Y, Wang T, Wang P, Zhang H, Zhang Q, et al The presence of polychlorinated biphenyls in yellow pigment products in China with emphasis on 3,3'-dichlorobiphenyl (PCB 11). Chemosphere. 2014;98:44–50. 10.1016/j.chemosphere.2013.09.075 24231041

[56] Shang H, Li Y, W Thanh, Wang P, Zhang H, Zhang Q, et al The presence of polychlorinated biphenyls in yellow pigment products in China with emphasis on 3,3 '-dichlorobiphenyl (PCB 11). Chemosphere. 2014;98:44–50. 10.1016/j.chemosphere.2013.09.075 24231041

[57] Choi S-D, Baek S-Y, Chang Y-S, Wania F, Ikonomou MG, Yoon Y-J, et al Passive air sampling of polychlorinated biphenyls and organochlorine pesticides at the Korean Arctic and Antarctic research stations: Implications for long-range transport and local pollution. Environ Sci Technol. 2008;42(19):7125–31. 18939536 10.1021/es801004p

[58] Hu D, Martinez A, Hornbuckle KC. Sedimentary records of non-Aroclor and Aroclor PCB mixtures in the Great Lakes. J Gt Lakes Res. 2011;37(2):359–64. 10.1016/j.jglr.2011.03.001 PMC3607302 23538476

[59] Tornkvist A, Glynn A, Aune M, Darnerud PO, Ankarberg EH. PCDD/F, PCB, PBDE, HBCD and chlorinated pesticides in a Swedish market basket from 2005—levels and dietary intake estimations. Chemosphere. 2011;83(2):193–9. 10.1016/j.chemosphere.2010.12.042 21269658

[60] Schecter A, Päpke O, Harris TR, Tung KC, Musumba A, Olson J, et al Polybrominated diphenyl ether (PBDE) levels in an expanded market basket survey of U.S. food and estimated PBDE dietary intake by age and sex. Environ Health Perspect. 2006;114(10):1515–20. 10.1289/ehp.9121 17035135 PMC1626425

[61] Schecter A, Haffner D, Colacino J, Patel K, Papke O, Opel M, et al Polybrominated diphenyl ethers (PBDEs) and hexabromocyclodecane (HBCD) in composite U.S. food samples. Environ Health Perspect. 2010;118(3):357–62. 10.1289/ehp.0901345 20064778 PMC2854763

[62] Darnerud PO, Atuma S, Aune M, Bjerselius R, Glynn A, Grawe KP, et al Dietary intake estimations of organohalogen contaminants (dioxins, PCB, PBDE and chlorinated pesticides, e.g. DDT) based on Swedish market basket data. Food Chem Toxicol. 2006;44(9):1597–606. 10.1016/j.fct.2006.03.011 16730400

[63] Hale RC, Alaee M, Manchester-Neesvig JB, Stapleton HM, Ikonomou MG. Polybrominated diphenyl ether flame retardants in the North American environment. Environ Int. 2003;29(6):771–9. 10.1016/S0160-4120(03)00113-2 12850095

[64] Alaee M, Arias P, Sjodin A, Bergman A. An overview of commercially used brominated flame retardants, their applications, their use patterns in different countries/regions and possible modes of release. Environ Int. 2003;29(6):683–9. 10.1016/S0160-4120(03)00121-1 12850087

[65] Guardia MJL, Hale RC, H E.. Detailed polybrominated diphenyl ether (PBDE) congener composition of the widely used penta-, octa-, and deca-PBDE technical flame retardant mixtures. Environ SciTechnol. 2006;40:8. 10.1021/es060630m 17120549

[66] Parolini M, Guazzoni N, Binelli A, Tremolada P. Polybrominated diphenyl ether contamination in soil, vegetation, and cow milk from a high-mountain pasture in the Italian Alps. Arch Environ Contam Toxicol. 2012;63(1):29–44. 10.1007/s00244-012-9753-8 22402779

[67] Domingo JL, Marti-Cid R, Castell V, Llobet JM. Human exposure to PBDEs through the diet in Catalonia, Spain: temporal trend. A review of recent literature on dietary PBDE intake. Toxicology. 2008;248(1):25–32. 10.1016/j.tox.2008.03.006 18420330

[68] La AGMJ, Hale RC, Harvey E. Detailed polybrominated diphenyl ether (PBDE) congener composition of the widely used penta-, octa-, and deca-PBDE technical flame-retardant mixtures. Environ Sci Technol. 2006;40(20):6247–54. Epub 2006/11/24. 17120549 10.1021/es060630m

[69] Feo ML, Gross MS, McGarrigle BP, Eljarrat E, Barcelo D, Aga DS, et al Biotransformation of BDE-47 to potentially toxic metabolites is predominantly mediated by human CYP2B6. Environ Health Perspect. 2013;121(4):440–6. 10.1289/ehp.1205446 23249762 PMC3620761

[70] Brambilla G, Abate V, di Domenico A, Esposito M, Fulgenzi AR, Iacovella N, et al Non-dioxin-like PCB and PBDE deposition on Zea mays L. leaves: modelled contamination in milk from dairy animals fed on silage. Food Addit Contam Part A: Chem, Anal, Control. 2015;32(6):864–73. 10.1080/19440049.2015.1029993 25806654

[71] Sun J, Pan L, Chen J, Li K, Zhu L. Uptake, translocation, and metabolism of hydroxylated and methoxylated polychlorinated biphenyls in maize, wheat, and rice. Environ Sci Pollut Res Int. 2016. 10.1007/s11356-016-7724-8 27699658

[72] Wang S, Zhang S, Huang H, Zhao M, Lv J. Uptake, translocation and metabolism of polybrominated diphenyl ethers (PBDEs) and polychlorinated biphenyls (PCBs) in maize (Zea mays L.). Chemosphere. 2011;85(3):379–85. 10.1016/j.chemosphere.2011.07.002 21798573

[73] Lake IR, Foxall CD, Lovett AA, Fernandes A, Dowding A, White S, et al Effects of river flooding on PCDD/F and PCB levels in cows' milk, soil, and grass. Environ Sci Technol. 2005;39(23):9033–8. 16382922 10.1021/es051433a

[74] Gronewald JW, Aboukhalil W, Weber EJ, Hanson JB. Lipid-composition of a plasma membrande enriched fraction of maize roots. Phytochemistry. 1982;21(4):859–62.

[75] National Academies of Sciences E, Medicine. Nutrient requirtments of beef cattle: eighth revised edition Washington, DC: The National Academies Press; 2016 494 p. 38386771

[76] Alexander BM, Baxter CS. Flame-retardant contamination of firefighter personal protective clothing—A potential health risk for firefighters. J Occup Environ Hyg. 2016;13(9):D148–55. 10.1080/15459624.2016.1183016 27171467

[77] Park JS, Voss RW, McNeel S, Wu N, Guo T, Wang Y, et al High exposure of California firefighters to polybrominated diphenyl ethers. Environ Sci Technol. 2015;49(5):2948–58. 10.1021/es5055918 25643236

[78] Shaw SD, Berger ML, Harris JH, Yun SH, Wu Q, Liao C, et al Persistent organic pollutants including polychlorinated and polybrominated dibenzo-p-dioxins and dibenzofurans in firefighters from Northern California. Chemosphere. 2013;91(10):1386–94. 10.1016/j.chemosphere.2012.12.070 23395527

[79] Shen B, Whitehead TP, McNeel S, Brown FR, Dhaliwal J, Das R, et al High levels of polybrominated diphenyl ethers in vacuum cleaner dust from California fire stations. Environ Sci Technol. 2015;49(8):4988–94. 10.1021/es505463g 25798547

[80] Stapleton HM, Sharma S, Getzinger G, Ferguson PL, Gabriel M, Webster TF, et al Novel and high volume use flame retardants in US couches reflective of the 2005 PentaBDE phase out. Environ Sci Technol. 2012;46(24):13432–9. 10.1021/es303471d 23186002 PMC3525014

[81] Webb H, Arnott J, Crawford R, Ivanova E. Plastic degradation and its environmental implilcations with special reference to poly (ethylene terephthalate). Polymers. 2013;5(1):1.

